# Association between Hospital Carotid Endarterectomy Procedure Volumes and In-Hospital Mortality in São Paulo State

**DOI:** 10.1590/1677-5449.202201642

**Published:** 2023-09-18

**Authors:** Renato Luís Pessôa

**Affiliations:** 1 Universidade do Vale do Taquari - UNIVATES, Lajeado, RS, Brasil.

**Keywords:** carotid arteries, endarterectomy, mortality, health policy, vascular surgery, artérias carótidas, endarterectomia, mortalidade, política de saúde, cirurgia vascular

## Abstract

**Background:**

Previous studies indicate an inverse relationship between hospital volume and mortality after carotid endarterectomy. However, data at the level of Brazil are lacking.

**Objectives:**

To assess the relationship between hospital carotid endarterectomy procedure volumes and mortality in the state of São Paulo.

**Methods:**

Data from the São Paulo State Hospital Information System on all carotid endarterectomies performed between 2015 and 2019 were analyzed. Hospitals were categorized into clusters by annual volume of surgeries (1-10, 11-25, and ≥26). Multiple logistic regression models were used to determine whether the volume of carotid endarterectomy procedures was an independent predictor of in-hospital mortality among patients undergoing this procedure.

**Results:**

Crude in-hospital mortality was nearly 60 percent lower in patients who underwent carotid endarterectomy at the highest volume hospitals than among those who underwent endarterectomy at the lowest volume hospitals (unadjusted OR of survival to hospital discharge, 2.41; 95% CI, 1.11-5.23; p = 0.027). Although this lower rate represents 1.5 fewer deaths per 100 patients treated, high-volume centers are more likely than low-volume centers to perform elective procedures, thus the analysis did not retain statistical significance when adjusted for admission character (OR, 1.69; 95% CI, 0.74-3.87; p = 0.215).

**Conclusions:**

In a contemporary Brazilian registry, higher volume carotid endarterectomy centers were associated with lower in-hospital mortality than lower volume centers. Further studies are needed to verify this relationship considering the presence of symptoms in patients.

## Resumo

**Contexto:**

Estudos indicam uma relação inversa entre volume hospitalar e mortalidade após endarterectomia carotídea. Entretanto, não há dados a nível brasileiro.

**Objetivos:**

Avaliar a relação entre volume hospitalar de endarterectomia carotídea e mortalidade no estado de São Paulo.

**Métodos:**

Foram analisados dados do Sistema de Informação Hospitalar do Estado de São Paulo de todas as endarterectomias carotídeas realizadas entre 2015 e 2019. Os hospitais foram categorizados em grupos de acordo com o volume anual de cirurgias (1-10, 11-25 e ≥26). Modelos de regressão logística múltipla foram usados para determinar se o volume de endarterectomias carotídeas era um preditor independente de mortalidade intra-hospitalar entre os pacientes submetidos a esse procedimento.

**Resultados:**

A mortalidade intra-hospitalar foi quase 60% menor nos pacientes submetidos a endarterectomia carotídea nos hospitais de maior volume em comparação aos pacientes submetidos a endarterectomia nos hospitais de menor volume (OR não ajustado de sobrevida após alta hospitalar, 2,41; IC 95%, 1,11-5,23; *p* = 0,027). Embora essa taxa mais baixa represente 1,5 menos mortes por 100 pacientes tratados, os centros de alto volume são mais propensos do que os centros de baixo volume a realizarem procedimentos eletivos; portanto, a análise não reteve significância quando ajustada para o caráter de admissão (OR, 1,69; IC 95%, 0,74-3,87; p = 0,215).

**Conclusões:**

Em um registro brasileiro contemporâneo, centros com maior volume de endarterectomia carotídea foram associados a menor mortalidade intra-hospitalar em comparação aos centros de menor volume. Mais estudos são necessários para verificar essa relação considerando a presença de sintomas em pacientes.

## INTRODUCTION

Carotid stenosis is responsible for about 10 to 15 percent of all strokes, mainly due to carotid atherosclerosis, which is the most frequent mechanism of carotid obstruction. In carotid disease, the purpose of revascularization is stroke prevention.^1^ The carotid endarterectomy (CEA) is a widely studied procedure that has been practiced for over 60 years for severe stenosis.^2^ However, the role of hospital CEA procedure volume in mortality after the procedure is still not well defined in the current literature.

Many studies have documented an inverse relation between the rate of mortality and the number of CEA procedures performed by individual surgeons or hospitals.^3-6^ A large systematic review by Holt et al. included 25 studies with nearly 900,000 CEAs and assessed the effect of hospital volume on outcomes, showing that the rate of post-operative complications (including mortality) decreased as the annual hospital volume of CEAs increased.^7^ The best surgical outcomes were observed when CEA was performed in hospitals with a critical annual volume threshold of 79 procedures. Most of the studies assessed were based on administrative data from hospitals located in the United States and European countries. However, the authors suggested that volume thresholds should be analyzed and adapted to the characteristics of each health care system in an individualized manner to reduce complications following CEA procedures.

Therefore, to determine whether a higher volume of patients undergoing endarterectomy is associated with better outcomes in Brazilian Unified Health System (SUS) settings, we analyzed in-hospital mortality in contemporary practice across an entire state.

## METHODS

### Data ascertainment

The Hospital Information System (SIH) of the São Paulo Health Department (SES/SP) is a registry developed to record all services from hospitalizations financed by SUS by collecting hospital admission authorizations. Data were collected from all carotid endarterectomy procedures between January 2015 and December 2019.

### Study variables and primary outcome

The study variables included information on the hospital, patient demographics (gender and age group), mean time in the ICU (days), and characteristics of admission. The primary outcome was in-hospital mortality. The character of the admission (elective or urgent) was collected secondarily to the database and denotes how the admission report was issued, but is not a reliable indication of the presence or absence of symptoms in the patient.

In addition, no data on strokes after the procedures were available in the database and this outcome was therefore not evaluated.

### Hospital volume

Volume was calculated as the total number of patients who underwent carotid endarterectomy at each hospital divided by the total number of years for which the hospital reported data to the SIH. To minimize possible biases, hospitals that only performed one procedure over the 5 years analyzed were excluded. Since there is no evidence-based categorization of hospital volume, hospitals were categorized empirically into three clusters based on the thresholds from a recent study conducted in Germany by Kuehnl et al.,^6^ which examined the association between hospital volume of endarterectomies and the risk of death or stroke. Kuehnl et al. used two further cut-off points (47 to 79 and >80 annual procedures), however these cut-off points were not compatible with our data.

### Statistical analysis

Categorical variables were presented as absolute and relative frequencies and were compared using the chi-square test. The normality assumption for continuous variables was assessed using skewness and kurtosis values as well as graphic methods. Non-normally distributed continuous variables were presented as medians and quartiles. Numbers of days of ICU stay were presented as mean and SD and compared using Scheffe’s test.

Sequential binary logistic regressions accounting for hospital clusters were performed to study the relationship between volume and mortality. An additional analysis was conducted using volume as a continuous variable and the Pearson coefficient was applied to assess the correlation with mortality. All tests were two-tailed and final p values < 0.05 were considered statistically significant. All statistical analyses were performed using SPSS v.21 software.

### Ethics committee

In accordance with Brazilian National Health Council Resolution number 466 from December 2012, as the database used is of public domain, free and unrestricted access, without individual identification of the patients, Research Ethics Committee approval was not necessary.

## RESULTS

From 2015 to 2019, 2075 CEA procedures were recorded in the database. The annual volume of CEA procedures ranged from 1 to 52. After excluding patients treated at hospitals that performed just a single procedure (n = 4), 2071 CEA procedures were retained for further analysis. A total of 39 hospitals were analyzed, with a median annual volume of 7 surgical procedures (Q25-Q75, 2-14) (Table 1).

**Table 1:** Volume classification, number of hospitals providing carotid endarterectomy, number of patients, and hospital annual volume.

**Hospital ranking**	**Hospitals, n**	**Patients, n**	**Deaths, n**	**Annual hospital carotid endarterectomy volume**	**Annual hospital volume within the cluster, median (Q25-Q75)**
Low	26	452	13	1 to 10	3 (2-7)
Medium	7	550	11	11 to 25	14 (11-18)
High	6	1069	13	>26	30 (29-45)
Total	39	2071	37		

Q25 25% percentile, Q75 75% percentile.

Baseline characteristics of the study population are shown in Table 2. The average ICU stay in the cohort was 2.5 (SD, 1.2) days. Further investigation with Scheffe’s test revealed no significant difference in mean ICU stay between low and medium-volume vs. high-volume groups (*p* = 0.55-0.71).

**Table 2:** Baseline and patient admission characteristics by hospital carotid endarterectomy volume.

**Characteristics**	**Low (n=452)**	**Medium (n=550)**	**High (n=1069)**	***p* value**
Female sex	142 (31.4)	214 (38.9)	365 (34.1)	0.037
Age				0.627
<60y	65 (14.4)	88 (16.0)	139 (13.0)	
60 to 69y	174 (38.5)	208 (37.8)	444 (41.5)	
70 to 79y	185 (40.9)	219 (39.8)	415 (38.8)	
≥80y	28 (6.2)	35 (6.4)	71 (6.6)	
Admission character				<0.001
Urgent	181 (40.0)	180 (32.7)	139 (13.0)	
Elective	271 (59.9)	370 (67.3)	930 (87.0)	
Days in ICU, mean (SD)	2.6 (1.3)*	2.6 (0.8)**	2.0 (1.4)	*0.550
				**0.714

* Indicates the p value for Low compared to High. **Medium compared to High. ICU Intensive Care Unit, SD Standard Deviation.

**Figure 1:**
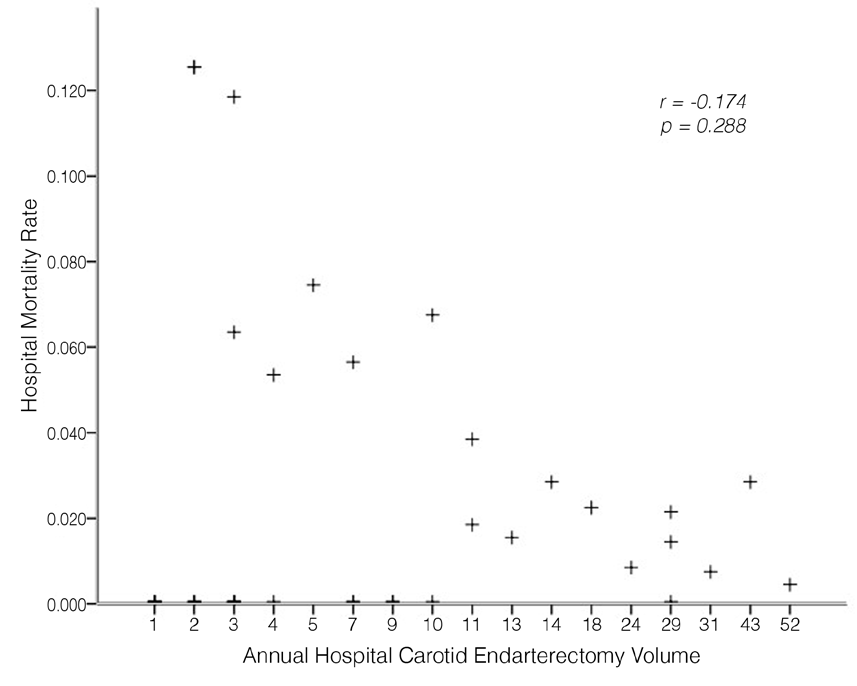
Scatterplot showing the relationship between carotid endarterectomy volume and in-hospital mortality (Pearson correlation coefficient and *p* value for correlation are shown).

The overall in-hospital mortality was 1.8% (incidence rate, 2.9% vs. 2.0% vs. 1.2% for low, medium, and high-volume centers respectively). In the unadjusted analysis, the odds of a good outcome were higher in high-volume hospitals compared to low-volume hospitals (OR, 2.41; 95% CI, 1.11-5.23; *p* = 0.027). The results were similar when the analysis was adjusted for demographic characteristics, but statistical significance was not maintained after adjustment for admission character (Table 3). Furthermore, there was no significant relationship between volume as a continuous variable and mortality (Pearson correlation coefficient = -0.174, *p* = 0.288) (Figure 1).

**Table 3:** Unadjusted and adjusted odds ratios in favor of survival to hospital discharge based on carotid endarterectomy volume.

**Model**	**High- vs. Low-Volume Hospitals (95%CI)**	***p* value**	**High- vs. Medium-Volume Hospitals (95%CI)**	***p* value**	**High- vs. Low- and Medium-Volume Hospitals (95%CI)**	***p* value**
**Unadjusted**	2.41 (1.11-5.23)	0.027	1.66 (0.74-3.73)	0.221	1.99 (1.01-3.94)	0.047
**Adjusted for demographics**	2.44 (1.12-5.31)	0.025	1.58 (0.70-3.56)	0.274	1.98 (1.00-3.92)	0.049
**Adjusted for demographics and admission character**	1.69 (0.74-3.87)	0.215	1.14 (0.49-2.66)	0.759	1.40 (0.69-2.86)	0.356

CI = Confidence Interval.

Other thresholds were tested a priori, but none of them achieved statistical significance. For example, using thresholds of 17 or fewer procedures per year, 18 to 28 procedures per year, and 29 or more procedures per year yielded mortality rates of 2.7% vs. 1.6% vs. 1.2% for low, medium, and high-volume hospitals, respectively. Multivariate adjusted ORs were 1.48 (95% CI, 0.69-3.20, *p* = 0.31) and 1.09 (95% CI, 0.37-3.15, *p* = 0.88) for high-volume compared with low and medium-volume hospitals, respectively.

## DISCUSSION

The primary finding of this large observational analysis was that in-hospital mortality was nearly 60 percent lower among patients who underwent CEA at hospitals with the highest volumes of such surgeries than among those who underwent endarterectomy at hospitals with the lowest volume. Despite this lower rate, which represented 1.5 fewer deaths per 100 patients, high-volume hospitals tended to perform a larger percentage of elective procedures, thus when adjusted for admission character, the analysis had no significant result. Some studies have indicated improved outcomes, despite adjustments for emergency admissions, however the volume threshold used was higher.^3,6^ Although some studies have suggested better outcomes with even higher volume thresholds, the most consistent cut-off-point is 79 CEA procedures.^6-8^ However, we did not employ this threshold in our study since the highest annual volume observed in any of the hospitals was 52 CEAs.

Notwithstanding, it should be noted that the data used are secondary and it was not possible to control whether all patients admitted as emergencies underwent emergency surgery or whether they had strokes or transient ischemic attacks. In addition, one of the variables that is associated with higher in-hospital mortality is presence of recent symptoms.^2,9^ Operating on more symptomatic patients could be linked with the higher mortality in hospitals with lower volume. However, the admission character recorded does not necessarily imply symptomatic or urgently operated patients. It should therefore be considered that the analysis adjusted for admission character may be influenced by a registration bias.

Referral bias may be another reason why the analysis dropped in significance after adjusting for emergency admissions. This type of bias can occur when a physician’s or a hospital’s reputation for superior outcomes leads to increased referrals, including referrals of patients with minor disorders, who may have had superior outcomes regardless of treatment.^10^ Therefore, it is possible that differences in transfer rates play a role in the observed heterogeneity in emergency department admission rates between patients who underwent endarterectomy at high-volume hospitals and those who underwent endarterectomy at low-volume hospitals.

No randomized trials have evaluated the effect of different carotid endarterectomy volumes on outcome. Most studies are multicenter, retrospective analyses with significant methodological differences. Although randomization of CEA to high-volume versus low-volume centers would appear to run into certain practical problems, a causal relationship between hospital CEA volume and treatment outcome cannot be inferred from observational studies. Nevertheless, these studies are reasonably informative.

Moreover, a prospective cohort associated prolonged postoperative stay after CEA with increased risk of readmission and long-term mortality.^11^ Meanwhile, in the present study, low-volume hospitals had no significant difference in mean length of ICU stay compared to high-volume hospitals. Thus, consequences of prolonged ICU stay did not differ significantly between the clusters.

For primary analysis, hospitals conducting just one procedure over the 5 years analyzed were excluded. This was performed to minimize selection bias and to account for hospitals that had participated for a short time. Nevertheless, even inclusion of these patients would not have changed the results. Unsurprisingly, most of the present cohort was made up of men and individuals over the age of 60, both of which are risk factors for carotid occlusion. In published Brazilian cohorts, a high prevalence of smoking (66-76%) has also been described in patients with significant carotid obstruction. This clinical feature could not be assessed with the data available in the database used, but it is likely that these numbers would be similar in this cohort.^12,13^

The study has some limitations that should be noted: its observational design and the possibility of residual confounding. Meanwhile its strengths include a large sample and adjustment for important potential confounders. In addition, considering that stroke is the main complication related to carotid endarterectomy surgeries, the unavailability of this data is a major limitation of the study.

## CONCLUSIONS

In a contemporary Brazilian registry, higher volume carotid endarterectomy centers were associated with lower in-hospital mortality compared to lower volume centers. Future studies should examine this relationship seeking to adjust for patients with symptomatic versus asymptomatic carotid disease.
